# *In vivo* multimodal imaging of adenosine A_1_ receptors in neuroinflammation after experimental stroke

**DOI:** 10.7150/thno.51046

**Published:** 2021-01-01

**Authors:** Ana Joya, María Ardaya, Alejandro Montilla, Maider Garbizu, Sandra Plaza-García, Vanessa Gómez-Vallejo, Daniel Padro, Juan José Gutiérrez, Xabier Rios, Pedro Ramos-Cabrer, Unai Cossío, Krishna R Pulagam, Makoto Higuchi, María Domercq, Fabio Cavaliere, Carlos Matute, Jordi Llop, Abraham Martín

**Affiliations:** 1 Achucarro Basque Center for Neuroscience, 48940 Leioa, Spain.; 2 CIC biomaGUNE, Basque Research and Technology Alliance (BRTA), Paseo Miramon 182, 20014, San Sebastian, Spain.; 3 Department of Neuroscience, University of Basque Country (UPV/EHU) and CIBERNED, 48940 Leioa, Spain.; 4 Departamento de Química Física, Facultad de Ciencia y Tecnología, Universidad del País Vasco (UPV/EHU), 48940, Leioa, Spain.; 5 Ikerbasque Basque Foundation for Science, 48013 Bilbao, Spain.; 6 National Institute of Radiological Sciences, National Institutes for Quantum and Radiological Science and Technology, Chiba, Japan.; 7 Centro de Investigación Biomédica en Red - Enfermedades Respiratorias, CIBERES, 28029 Madrid, Spain.

**Keywords:** [^18^F]CPFPX, [^18^F]DPA-714, [^18^F]FLT, PET, A_1_ARs, cerebral ischemia, MRI, neuroinflammation

## Abstract

Adenosine A_1_ receptors (A_1_ARs) are promising imaging biomarkers and targets for the treatment of stroke. Nevertheless, the role of A_1_ARs on ischemic damage and its subsequent neuroinflammatory response has been scarcely explored so far.

**Methods:** In this study, the expression of A_1_ARs after transient middle cerebral artery occlusion (MCAO) was evaluated by positron emission tomography (PET) with [^18^F]CPFPX and immunohistochemistry (IHC). In addition, the role of A_1_ARs on stroke inflammation using pharmacological modulation was assessed with magnetic resonance imaging (MRI), PET imaging with [^18^F]DPA-714 (TSPO) and [^18^F]FLT (cellular proliferation), as well as IHC and neurofunctional studies.

**Results:** In the ischemic territory, [^18^F]CPFPX signal and IHC showed the overexpression of A_1_ARs in microglia and infiltrated leukocytes after cerebral ischemia. Ischemic rats treated with the A_1_AR agonist ENBA showed a significant decrease in both [^18^F]DPA-714 and [^18^F]FLT signal intensities at day 7 after cerebral ischemia, a feature that was confirmed by IHC results. Besides, the activation of A_1_ARs promoted the reduction of the brain lesion, as measured with T_2_W-MRI, and the improvement of neurological outcome including motor, sensory and reflex responses. These results show for the first time the *in vivo* PET imaging of A_1_ARs expression after cerebral ischemia in rats and the application of [^18^F]FLT to evaluate glial proliferation in response to treatment.

**Conclusion:** Notably, these data provide evidence for A_1_ARs playing a key role in the control of both the activation of resident glia and the *de novo* proliferation of microglia and macrophages after experimental stroke in rats.

## Introduction

Cerebral ischemia triggers an acute increase in the concentration of purines (adenosine 5'-triphosphate-ATP and adenosine) that act as extracellular signaling molecules through a large variety of metabotropic P1 adenosine receptors (A_1_, A_2A_, A_2B_ and A_3_), metabotropic P_2_Y and ionotropic P_2_X purinoreceptors 1, 2. Adenosine receptors are expressed in the central nervous system, where they play a crucial role in a wide variety of physiological responses such as learning, memory, locomotor activity and vasodilation, among others 3, 4. In addition, adenosine receptors have shown to be broadly expressed in both the innate and adaptive immunity, suggesting its control on the neuroinflammatory response 5. Following ischemic brain injury, A_1_ adenosine receptors (A_1_ARs) modulate the release of IL-10 by immune cells, and the infusion of adenosine to the rat brain has shown protective effects reducing the infarct volume and improving the neurological outcome 6-10. The temporal evolution of the expression of A_1_ARs after cerebral ischemia remains unknown and hence, the use of *in vivo* imaging modalities would be extremely helpful to gain knowledge on their expression and the protective role that they might play. During the last two decades, radiotracers such as 8-cyclopentyl-3-(3-[^18^F]fluoropropyl)-1-propylxanthine ([^18^F]CPFPX) have been developed for non-invasive positron emission tomography (PET) imaging of adenosine A_1_ARs in both human and rodent living brains 11, 12. [^18^F]CPFPX is a fluorinated analogue of 8-cyclopentyl-1,3-dipropylxanthine (DPCPX), considered as the prototypical A_1_ARs antagonist due to its high selectivity and affinity for these receptors 13. In addition, [^18^F]CPFXP has shown rapid blood brain barrier penetration, a cerebral binding proportional to the distribution of these receptors and a reproducible and reliable non-invasive quantification of A_1_AR density in the rat brain 12, 14. Nevertheless, although PET imaging studies have reported alterations of A_1_AR density in neurological diseases 15-18, the role of these receptors on neuroinflammation, as revealed by nuclear imaging is still largely unknown. For this reason, this study aims to investigate the relationship of A_1_ARs with the neuroinflammatory reaction that follows stroke using multimodal imaging methods, immunohistochemistry and neurofunctional evaluation after cerebral ischemia in rats. Ischemic rats treated with the selective A_1_AR antagonist (DPCPX) and the highly selective agonist (ENBA) were subjected to PET studies with [^18^F]DPA-714, an specific radioligand for the translocator protein (18kDa) (TSPO), and [^18^F]FLT for the evaluation of microglia/macrophage activation and proliferation 19-23. TSPO overexpression has been used as a surrogate marker for inflammatory activation after ischemia 20, 24, while a recent work form our group has demonstrated that [^18^F]FLT PET is able to detect proliferative inflammatory cells in the ischemic area after stroke 25. Finally, the pharmacological modulation of A_1_ARs and its effects on brain damage was studied with magnetic resonance imaging (MRI) and neurological evaluation.

The results reported here provide novel information about the role of A_1_ARs on stroke outcome and the subsequent inflammatory reaction in rats. *In vivo* imaging of the therapeutic and diagnostic potential of adenosine receptors is a promising strategy for stroke care, since adenosine signaling exerts control on the ischemic damage and neuroinflammation after cerebral ischemia 26. Hence, the establishment of novel biomarkers such as adenosine receptors can contribute to accelerate the development of novel therapies for stroke 27.

## Materials and Methods

### Cerebral ischemia in rats

8-weeks old -male Sprague-Dawley rats (n = 85; 304 ± 7.1 g body weight; Janvier, France) were used for both non-invasive imaging, metabolism and immunohistochemical studies. Animal experimental protocols and relevant details regarding welfare were approved by our institutional animal care and use committee (IACUC) and local authorities (Diputación Foral of Guipuzcoa) and were conducted in accordance with the ARRIVE guidelines and Directives of the European Union on animal ethics and welfare. All studies were conducted in our AAALAC certified SPF facilities.

Rats were anaesthetized with 2.5% isoflurane in 100% O_2_ and the right common, external, and internal carotid arteries were exposed through a ventral cervical midline incision. After electrocoagulation, the external carotid artery was ligated and cut 3-5 mm distal to the bifurcation. The pterygopalatine artery was ligated, and micro-clips were placed across both the common and external carotid arteries. A 2.6-cm length of 4-0 monofilament nylon suture (Sutures Aragó, Barcelona) heat-blunted at the tip was introduced into the external carotid artery through a puncture and gently advanced into the internal carotid artery and circle of Willis until the origin of the MCA was reached. During occlusion, animals were allowed to recover in their cages for 80 min. 10 min before the end of the 90-min occlusion, animals were re-anaesthetized, the filament and the clip of the common carotid artery were gently removed and rats were kept in their cages with free access to food and water.

### Experimental set-up and treatments

**Figure 1:**
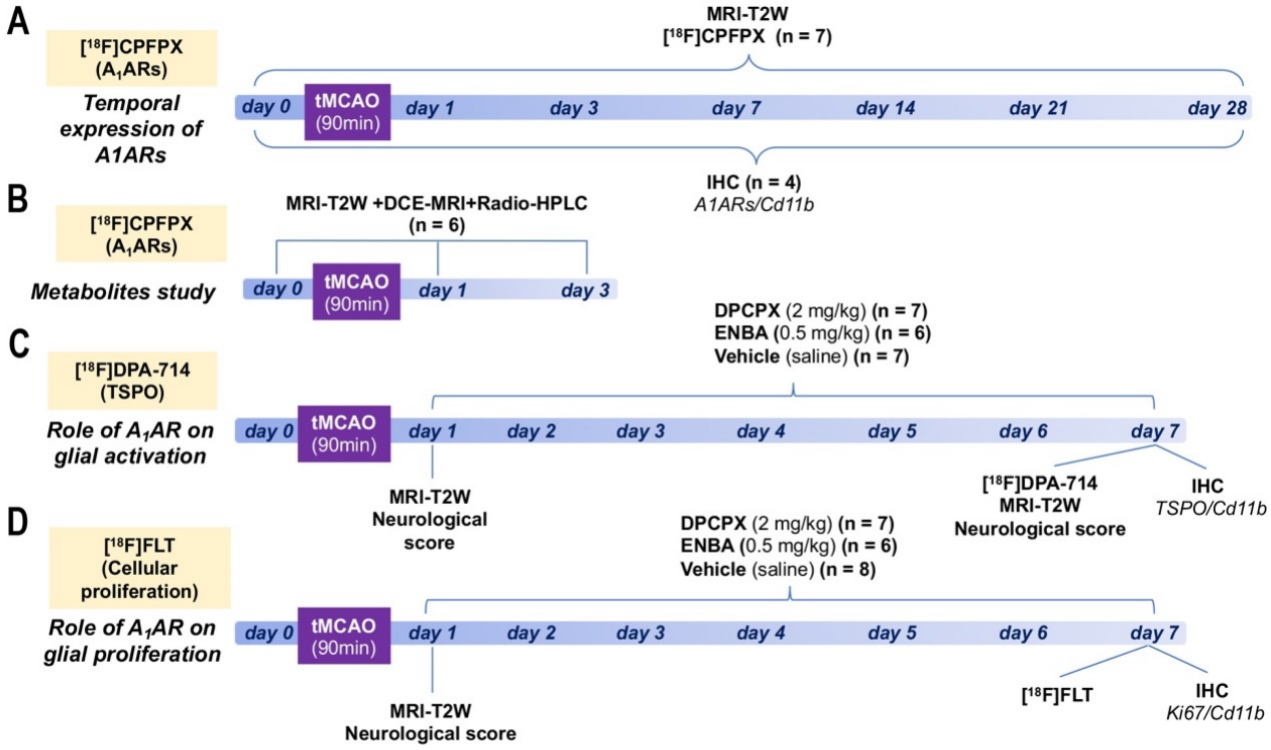
Experimental set-up of the imaging and treatments studies. Temporal expression of A_1_ARs (A) and metabolite analysis of [^18^F]CPFPX after cerebral ischemia. Evaluation of the role of A_1_ARs receptors on glial activation (B) and proliferation (C).

Ischemic rats were subjected to T_2_-weighted (T_2_W) MRI scans at 24 h after reperfusion to select rats presenting cortico-striatal infarction for inclusion in the PET studies. 7 rats were repeatedly scanned by PET before reperfusion (day 0) and at 1, 3, 7, 14, 21 and 28 days after ischemic onset to evaluate the binding of A_1_ARs (Figure 1A). Subsequently, a group of 6 rats were subjected to MRI-T_2_W and Dynamic Contrast Enhanced (DCE)-MRI to evaluate brain lesion, the blood-brain barrier (BBB) disruption (BBBd) and radio-HPLC for metabolite analysis of [^18^F]DPCPX at control and days 1 and 3 after ischemia (Figure 1B). During 6 consecutive days, starting at day 1 following MCAO, a first group of 14 rats was treated daily with intraperitoneal (i.p.) administration of 8-cyclopentyl-1,3-dipropylxanthine (DPCPX, selective A_1_AR antagonist; 0.1 mL; 1.25 mg/kg), a second group of 12 rats was treated daily with i.p. administration of (±)-5'-chloro-5'-deoxy-ENBA (ENBA, highly selective A_1_AR agonist; 0.3 mL; 0.5 mg/kg, i.p.) and a third control ischemic group of 18 rats received the same daily volume of vehicle (saline) in a randomized and blinded fashion (Figures 1C and D). At day 7, all rats from these 3 groups were imaged with PET and MRI to determine the effect of DPCPX and ENBA on TSPO expression, glial proliferation, brain lesion volume and neurological outcome. *Ex vivo* ICH studies for TSPO and glial proliferation were also performed on those animals to validate PET imaging findings. Finally, 28 additional rats were used to perform IHC for A_1_ARs expression at 0, 1, 3, 7, 14, 21 and 28 days after cerebral ischemia (n = 4 per time point).

### Magnetic resonance imaging

T_2_W-MRI scans were acquired for rats subjected to the PET studies, to evaluate the infarction volume and to show the stroke evolution over the first month after ischemia onset. Furthermore, T_2_W-MRI scans were used to evaluate the infarction volume in treated and control rats before (day 1) and after the treatment (day 7). In addition, Dynamic Contrast Enhanced (DCE)-MRI scans at control and days 1-3 after ischemia were carried out to assess the influence of BBBd on the metabolite accumulation in the brain. Scans were performed in rats anaesthetized with isoflurane (2-2.5%) in a 30/70% mixture of O_2_/N_2_. Animals were placed into an MRI rat compatible holder and maintained in normothermia using a water-based heating blanket at 37 °C. To ensure animal welfare, temperature and respiration rate were continuously monitored while they remained in the MRI magnet, using a SAII M1030 system (SA Instruments, NY, USA). MRI *in vivo* studies were performed on a 7T horizontal bore Bruker Biospec USR 70/30 MRI system (Bruker Biospin GmbH, Ettlingen, Germany), interfaced to an AVANCE III console, and with a BGA12-S imaging gradient insert (maximal gradient strength 400 mT/m, switchable within 80 µs). Measurements were performed with a 72 mm volumetric quadrature coil for excitation and a 20 mm rat brain surface coil for reception. The imaging session started with the acquisition of a scout scan, which was used to plan the whole study focusing on the region of interest. T_2_W images were acquired with a Bruker's RARE (Rapid Acquisition with Relaxation Enhancement) sequence (Effective TE = 40 ms, TR = 4400 ms, NA = 2; Matrix = 256 × 256 points; FOV = 25.6 × 25.6 mm; spatial resolution = 100 × 100 µm; 24 contiguous slices of 1 mm thickness covering the whole brain), which was used to quantify the volume of the lesion.

For the evaluation of BBB integrity, the tail vein was catheterized with a 24-gauge catheter for intravenous administration of the contrast agent (Multihance, 0.2 mmol/ml, 1 ml/Kg body weight). The BBBd was assessed using DCE-MRI, which allowed for differentiation between changes in vascular permeability and changes in the extravascular extracellular space (EES). The T1-weighted (T_1_W) DCE-MRI was acquired with a Bruker's FLASH (Fast low-angle shot) sequence (TE = 2.5 ms; TR = 31.25 ms; FA = 15º; NA = 1; NR = 200; Matrix = 128 × 128 points; FOV = 25.6 × 25.6 mm; spatial resolution = 200 × 200 µm; 5 slices of 1 mm thickness covering the ischemic lesion). This technique involves the serial acquisition in rapid succession of MR images (NR = 200) of a tissue of interest, being the ischemic lesion in this particular case, before and after the intravenous administration of Multihance, a gadolinium-based paramagnetic contrast agent. DCE-MRI allows for monitoring changes in the T1 relaxation rate of the tissue after the administration of gadolinium with repeated serial imaging using T_1_W images.

### Magnetic resonance imaging analysis

For image analysis, regions of interest (ROIs) were manually defined using the open source software 3D Slicer (Version 4.8 http://www.slicer.org). The calculation of the lesion volume using MRI was carried out by summing the areas of infarcted regions showing hyperintense signals of all slices affected by the lesion. BBB Permeability (ktrans) maps were obtained from DCE-MRI images with DCE@urLAB software 28 in the lesion volume measured with T_2_W-MRI, using the Tofts Model with the following input parameters: T10 (tissue) pixel-wise obtained from acquired T1 maps, T10 blood = 2070 ms 29, TR = 31.25 ms, FA = 15º, frame period = 2s, Nº frames = 200, contrast agent relaxivity = 4.24 mM-1 s-1, hematocrit = 0.43, Injection frame, Frames for IAUC= 5 and Arterial Input Function (AIF) as measured from a region of interest located at the carotid artery.

### Radiochemistry

The production and quality control of [^18^F]DPA-714 (non-decay corrected radiochemical yield = 11 ± 2%; radiochemical purity > 95% at the time of injection; molar activity values 160-430 GBq/µmol at the end of the synthesis) and [^18^F]FLT (non-decay corrected radiochemical yield = 8±1%; radiochemical purity > 95% at injection time) were carried out following established protocols in our laboratory 25. [^18^F]-8-Cyclopentyl-3-(3-fluoropropyl)-1-propylxanthine ([^18^F]CPFPX) was produced following a previously described method with minor modifications. In brief, dried ^18^F was reacted with the appropriate precursor ((8-cyclopentyl-3-(3-tosyloxypropyl)-7-pivaloyloxymethyl-1-propylxanthinethe, prepared as previously described; 30 2 mg) in dimethylsulfoxide (DMSO; 0.5 mL) (85 °C; 10 min). After cooling to room temperature, hydrolysis was conducted by addition of 2M sodium hydroxide aqueous solution (200 µL) (3 min, 30 °C). The reaction crude was neutralized with acetic acid (7 mmol), acetonitrile/water (1 mL; 60/40, v/v) was added and the mixture was purified by HPLC using a VP125/10 Nucleosil 100-7 C18 column (Macherey-Nagel, Düren, Germany) as the stationary phase and acetonitrile/water (60/40, v/v) as the mobile phase (flow rate = 7 mL/min). The desired fraction (t_R_ = 11-12 min) was collected, diluted with water (40 mL), and reformulated using a C-18 cartridge (Sep-Pak® Plus, Waters, Milford, MA, USA). The resulting ethanol solution (1 mL) was diluted with physiologic saline solution (9 mL) and filtered through 0.22 µm filter to yield the tracer was ready for injection. Chemical and radiochemical purity were determined by HPLC and co-elution with reference standard, prepared as previously described, 30 using a Mediterranean C18 column (4.6 × 250 mm, 5 µm; Teknokroma, Spain) as the stationary phase and aqueous KH_2_PO_4_ solution (3.3 g/L)/acetonitrile (30/70, v/v) as the mobile phase at a flow rate of 1 mL/min (t_R_ = 4.5 min). Non-decay corrected radiochemical yield was 17 ± 3%. Radiochemical purity was always > 95% at the time of injection, and molar activity values were within the range 260-540 GBq/µmol at the end of the synthesis.

### Positron emission tomography scans and data acquisition

PET scans were performed using an eXplore Vista PET-CT camera (GE Healthcare, Waukesha, WI, USA). Scans were performed in rats anaesthetized with 2-2.5% of isoflurane in 100% O_2_ at a constant time of day in order to avoid changes in receptor expression due to circadian rhythmicity. Animals were placed into a rat holder compatible with the PET acquisition system and maintained in normothermia using a water-based heating blanket at 37 °C. To ensure animal welfare, temperature and respiration rate were continuously monitored while they remained in the PET camera, using a SAII M1030 system (SA Instruments, NY, USA). The tail vein was catheterized with a 24-gauge catheter for intravenous administration of the radiotracer. For longitudinal assessment of A_1_ARs (radiotracer: [^18^F]CPFPX), rats were scanned before and during the following month after ischemia. The radioactivity (min-max, 35.52-81.31 MBq; 0.11-1.76 nmol; Table S1) was injected concomitantly with the start of the PET acquisition. During the first hour dynamic images of the brain were acquired for 60 min using 31 frames (3 × 5, 3 × 10, 3 × 15, 3 × 30, 4 × 60, 4 × 120, 5 × 180, 6 × 300 s) in the 400-700 keV energetic window. For evaluation of treatments on TSPO binding after ischemia, [^18^F]DPA-714 (min-max, 40.70-87.69 MBq; 0.21-1.80 nmol; Table S2) was injected at the start of the PET acquisition and dynamic brain images were acquired for 30 min using 23 frames (3 × 5, 3 × 15, 4 × 30, 4 × 60, 4 × 120, 5 × 180 s). Finally, [^18^F]FLT (min-max, 48.33-60.81 MBq; Table S3) was used to evaluate glial proliferation after ischemia. After the uptake period of 30 min, rats were anesthetized and placed on the PET for 30 min brain static acquisition. After each PET scan, CT acquisitions were also performed (140 mA intensity, 40 kV voltage), to provide anatomical information of each animal as well as the attenuation map for the later PET image reconstruction. Dynamic and static acquisitions were reconstructed (decay and CT-based attenuation corrected) with filtered back projection (FBP) using a Ramp filter with a cutoff frequency of 0.5 mm^-1^.

### Positron emission tomography image analysis

PET images were analyzed using PMOD image analysis software (Version 3.5, PMOD Technologies Ltd, Zurich, Switzerland). For the analysis of PET signal, both PET images and an MRI (T_2_W) rat brain template from Pmod were separately manually co-registered to the CT of the same animal to generate a spatial normalization. Subsequently, MRI brain template was automatically co-registered to PET images. Two type of Volumes of Interest (VOIs) were established as follows: (1) A first set of VOIs was defined to study the whole brain [^18^F]CPFPX, [^18^F]DPA-714 and [^18^F]FLT PET signals. Whole brain VOIs were manually drawn in both the entire ipsilateral and contralateral hemispheres on slices of the MRI (T_2_W) rat brain template from the PMOD software. (2) A second set of VOIs was automatically generated in the cerebral cortex and the striatum by using the regions proposed by the PMOD rat brain template, to study the evolution of [^18^F]CPFPX PET signal in these specific regions in both the ipsilateral and contralateral cerebral hemispheres. For dynamic PET scans, the last three ([^18^F]CPFPX) or five ([^18^F]DPA-714) frames were used to calculate radiotracers uptake during the last 15 min. For static PET scans, the 30 min frame was used to quantify the [^18^F]FLT uptake. Average values in each ROI were determined and expressed as percentage of injected dose per cubic centimeter (%ID/cc).

### Metabolite analysis

Rats were injected intravenously (tail vein) with of [^18^F]DPCPX (76 ± 12 MBq) under a general anesthesia (2-2.5% isoflurane in pure 100%). At t = 60 min, blood samples (ca. 500 µL) were obtained by cardiac puncture and animals were immediately perfused with heparinized saline solution. After complete perfusion, the brain was harvested. Blood samples were processed to separate the plasma, which was diluted with an equal volume of acetonitrile. After mixing vigorously for 20 s, samples were centrifuged at 2000 g for 4 min. The liquid phase was separated from the precipitate by decantation and was injected into the HPLC system equipped with a radioactivity detector (Gabi, Raytest), and using a Mediterranea C18 column (4.6 x 150 mm, 5 µm; Teknokroma, Spain) as the stationary phase and aqueous KH_2_PO_4_ solution (3.3 g/L) (A)/acetonitrile (B) as the mobile phase (flow rate = 1 mL/min), with the following gradient: t = 0 min, 90% A; t = 1 min, 90% A; t = 10 min, 10% A; t = 14 min, 10% A; t = 15 min, 90% A; t = 19 min, 90% A (retention time of [^18^F]DPCPX: 9.2 min). For the determination of metabolites in the brain, the organ was divided in two hemispheres, which were separately homogenized in a glass homogenizer tube. Acetonitrile in ultrapure water (1:1; 500 µL) was added, the homogenate centrifuged (2000 g, 5 min at room temperature) and the supernatant analyzed by HPLC as above. In all cases, the percentage of non-metabolized parent compound was calculated as the ratio between the area under the peak corresponding to [^18^F]DPCPX, and the sum of the areas of all peaks in the chromatogram.

### Immunohistochemistry and cell counting

Immunohistochemistry staining was carried out at day 0 and at 1, 3, 7, 14 and 28 days after ischemia and at day 7 in animals treated with DPCPX, Vehicle and ENBA. The brain was removed, frozen and cut in 5-*μ*m-thick sections in a cryostat. Sections were fixed in 4% paraformaldehyde during 15 min, washed with phosphate-buffered saline (PBS) and incubated 5 min in NH_4_Cl, following by two PBS rinse and methanol-acetone (1:1) permeabilization during 5 min at -20 ºC. After PBS washing, samples were saturated with a solution of bovine serum albumin (BSA) 5%/Tween 0.5% in PBS during 15 min at room temperature, and incubated during 2 h at room temperature with primary antibodies BSA (5%)/Tween (0.5%) in PBS. The first set of sections were stained with for A_1_AR with rabbit anti-rat A_1_ (1:300; Alomone Labs, Israel) and for CD11b with mouse anti-rat CD11b (1:300; Serotec, Raleigh, NC, USA). The second set of sections were stained for TSPO with a rabbit anti-rat TSPO (NP155, 1:1000) and for CD11b. Finally, the third set of sections were stained for Ki67 with rabbit anti-rat Ki67 (1:400, AbCam, Cambridge, UK) and CD11b. Sections were washed (3 × 10 min) in PBS and incubated for 1 h at room temperature with secondary antibodies Alexa Fluor 488 goat anti-rabbit IgG and Alexa Fluor 594 goat anti-mouse IgG (Molecular Probes, Life Technologies, Madrid, Spain, 1:1000) in BSA 5%/Tween 0.5% in PBS, washed again (3 × 10 min) in PBS, and mounted with a prolong antifade kit with or without DAPI in slices (Molecular Probes Life Technologies, Madrid). Standardized images acquisition was performed with the Panoramic MIDI II automated digital slide scanner (3DHistech Ltd., Hungary) for A_1_AR, the Axio Observer Z1 (Zeiss, Le Pecq, France) for TSPO and the Leica SP8 microscope (Hospitalet de Llobregat, Spain) for Ki67 imaging. Cells were manually counted in ten representative and different fields at 40 × and 100 × magnifications by using Image J (Version 2.0.0-rc-69/11.52p, NIH) software.

### Neurological assessment

The assessment of neurological outcome induced by cerebral ischemia was based on a previously reported 9-neuroscore test 31. This test is a global neurological assessment that was developed to measure neurological impairments following stroke and assesses a variety of motor, sensory and reflex responses. Before imaging evaluations, four consecutive tests were performed at days 1 and 7 after ischemia in treated and control rats as follows: (a) spontaneous activity (moving and exploring = 0, moving without exploring = 1, not moving = 2); (b) left drifting during displacement (none = 0, drifting only when elevated by the tail and pushed or pulled = 1, spontaneous drifting = 2, circling without displacement or spinning = 3), (c) parachute reflex (symmetrical = 0, asymmetrical = 1, contralateral forelimb retracted = 2), and (d) resistance to left forepaw stretching (stretching not allowed = 0, stretching allowed after some attempts = 1, no resistance = 2). Total score could range from 0 (normal) to a 9 (highest handicap) point-scale.

### Statistical analyses

For longitudinal PET imaging studies, values of percentage of injected dose per cubic centimeter (%ID/cc) within each region and time point following cerebral ischemia were averaged and compared with the averaged values of every time point using repeated measures ANOVA followed by Tukey's multiple-comparison tests for post-hoc analysis. For longitudinal neurological assessment, the neurological outcome at days 0 and 1 after cerebral ischemia were averaged and compared with the averaged values of every time point using the same statistical method used in PET studies. Cellular expression of microglial and infiltrated leukocytes/A_1_ARs before (day 0) and at different days after ischemia were compared using one-way ANOVA followed by Tukey's multiple-comparison tests for post-hoc analysis. For treatment studies, infarct volume and neurological score differences before (1day after MCAO) and after treatments (7 days after MCAO) were averaged and compared using two-way ANOVA with Sidak's multiple comparison tests for post-hoc test. Additionally, the effects of the treatments in PET uptake, infarct volume, microglial/TSPO and microglial/Ki67 at day 7 after ischemia were compared using a one-way ANOVA followed by Bonferroni's multiple-comparison tests for post hoc analysis. The level of significance was regularly set at *P* < 0.05. Statistical analyses were performed with GraphPad Prism version 8 software.

## Results

**Figure 2:**
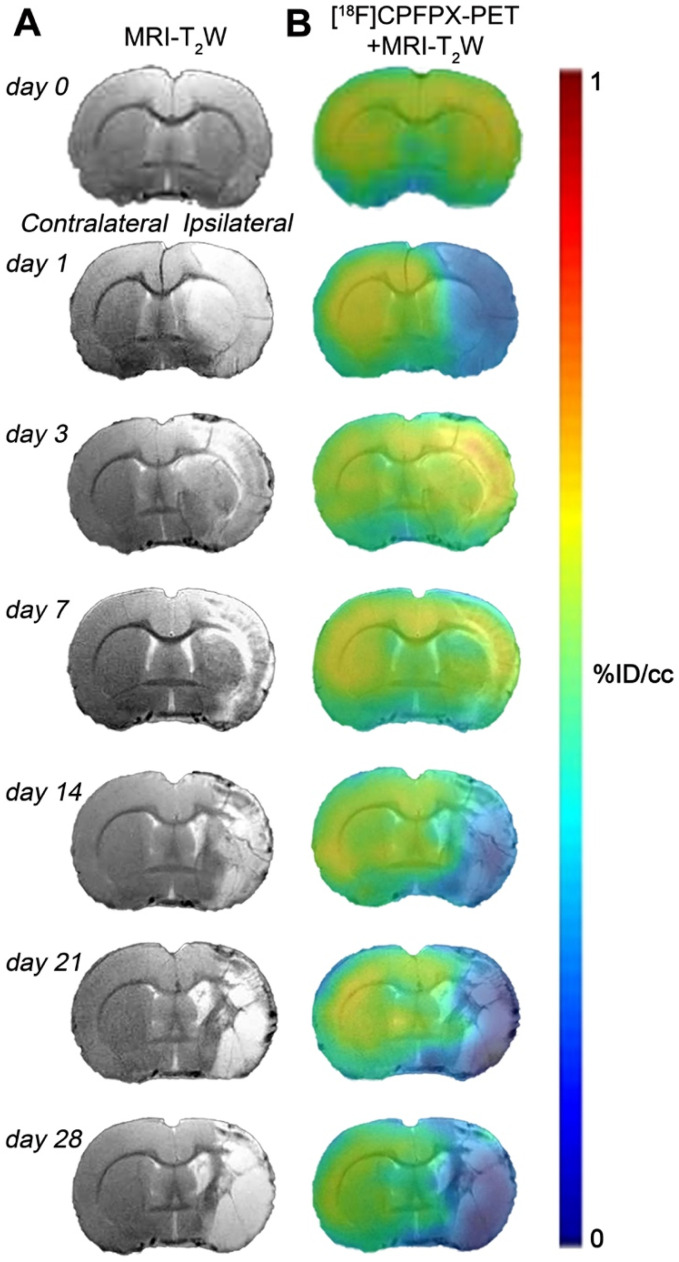
Magnetic resonance imaging (MRI) (T_2_-weighthing (T_2_W)) and Positron emission tomography (PET) images of [^18^F]CPFPX before (day 0) and at days 1, 3, 7, 14, 21 and 28 after cerebral ischemia in a representative rat. MRI-T_2_W (A) and co-registered [^18^F]CPFPX PET- MRI-T_2_W (B) axial images show the ischemic lesion evolution over one month after stroke onset.

Hyperintense regions of MRI-T_2_W images showed the formation of vasogenic edema as the result of the evolution of the brain ischemia at day 1 after 90 min MCAO followed by the progression of the lesion at 3, 7, 14, 21 and 28 days after ischemic stroke (Figure 2A). The extent of brain damage was assessed using T_2_W MRI at 1 day after ischemia (mean ± sd.: 326 ± 41 mm^3^, n = 7). PET images with normalized color scale illustrate the temporal expression and cerebral distribution of A_1_ARs with [^18^F]CPFPX in the rat brain at control and over the following month after ischemia stroke (Figure 2B).

### [^18^F]CPFPX after cerebral ischemia

**Figure 3:**
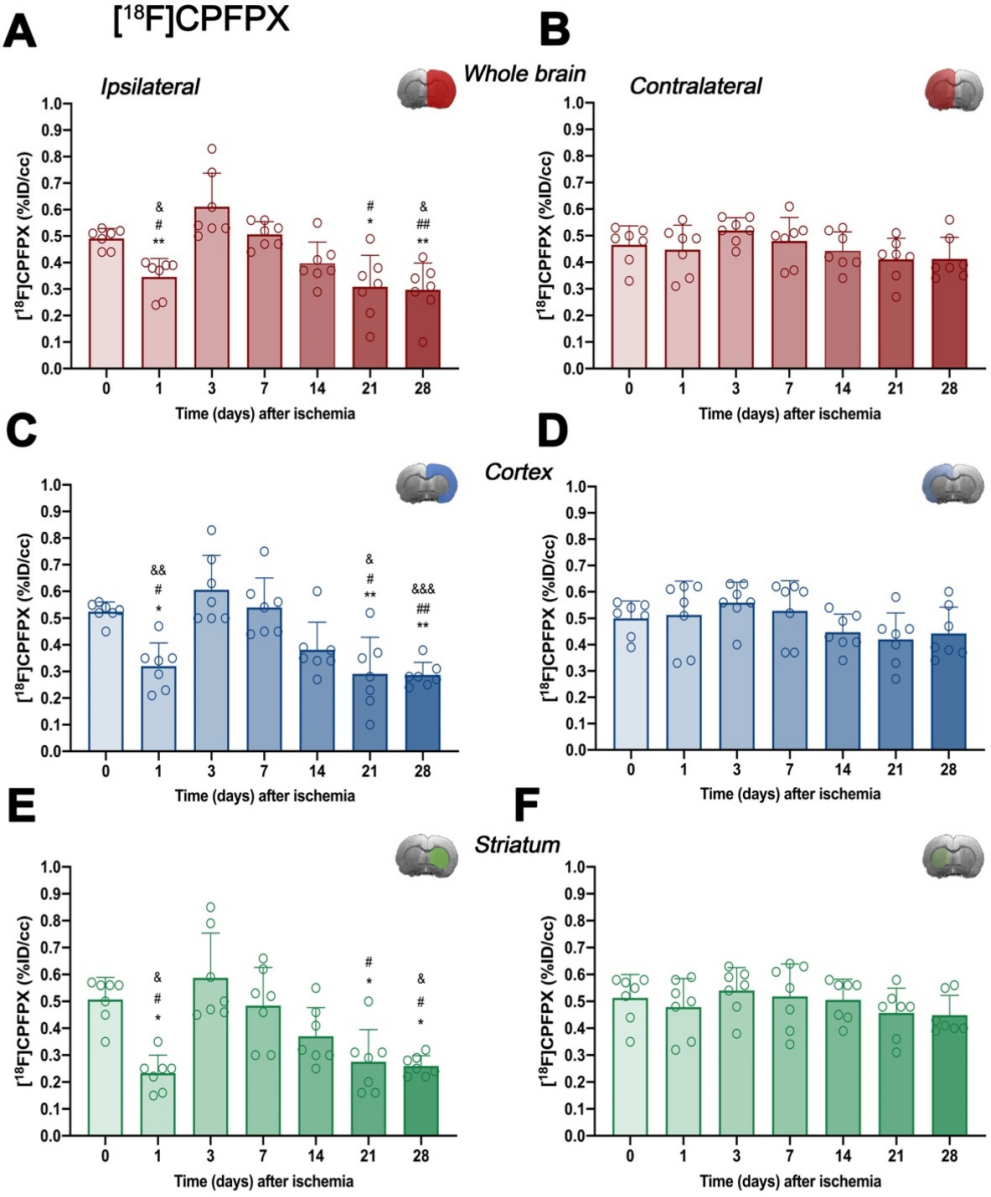
Time course of the progression of [^18^F]CPFPX PET signals (n = 7) before (day 0) and at different days after ischemia. %ID/cc of [^18^F]CPFPX was quantified in the entire ipsilateral cerebral hemisphere (A), contralateral hemisphere (B), ipsilateral cerebral cortex (C), contralateral cerebral cortex (D), ipsilateral striatum (E) and contralateral striatum (F). The upper right panel of each figure show the selected brain ROI for the quantification defined on an axial slice of a MRI (T_2_W) template. *p < 0.05 and **p < 0.01 compared with day 3; ^#^p < 0.05 and ^##^p < 0.01 compared with day 7; ^&^p < 0.05 and ^&&^p < 0.01 and ^&&&^p < 0.01 compared with day 0. [^18^F]CPFPX signal show non-significant changes at different days after MCAO in the contralateral hemisphere. Values are presented as scatter dot blot (mean ± SD).

*In vivo* imaging of A_1_ARs was evaluated with the radiotracer [^18^F]CPFPX in the ipsilateral and contralateral whole brain, cerebral cortex and striatum before (control) (67.80 ± 8.14 MBq; 0.37 ± 0.34 nmol) and at 1 (65.25 ± 13.11 MBq; 0.47 ± 0.29 nmol), 3 (64.48 ± 7.55 MBq; 0.56 ± 0.48 nmol), 7 (68.77 ± 7.62 MBq; 0.68 ± 0.58 nmol), 14 (66.37 ± 6.89 MBq; 0.61 ± 0.57 nmol), 21 (61.58 ± 12.46 MBq; 0.56 ± 0.52 nmol) and 28 (67.76 ± 3.56 MBq; 0.53 ± 0.56 nmol) days after MCAO (Figure 3 and Table S1). In the ipsilateral whole brain, [^18^F]CPFPX showed a significant decrease at day 1 after ischemia in comparison to baseline (*p* < 0.05, Figure 3A). Subsequently, an increase of PET signal was detected at days 3 (*p* < 0.001) and 7 (*p* < 0.05) with respect to day 1 after ischemia. The highest value was reached at day 3 after ischemia onset, compared with 14 (*p* < 0.01), 21 and 28 days (*p* < 0.001). In addition, PET signal significantly decreased at 21 and 28 days with respect to day 7 and baseline levels (*p* < 0.01; Figure 3A). In the contralateral whole brain, [^18^F]CPFPX signal showed similar values along the different days evaluated (Figure 3B). [^18^F]CPFPX PET signal at day 0 (control) showed similar values (circa 0.5 %ID/cc) in both cerebral cortex and striatum evidencing similar distribution of A_1_ARs in the main brain regions affected by MCAO (Figure 3C and E). Similarly to the whole brain, ischemic cerebral cortex and striatum showed a significant decrease (*p* < 0.01 versus baseline values) followed by a significant PET signal peak at day 3 (*p* < 0.01, versus baseline values and day 14) and followed by a progressive decrease from days 7 to 28 after cerebral ischemia. After the second week, PET values displayed similar values to those shown at day 1 after cerebral ischemia (Figures 3C and E). Finally, the contralateral cerebral cortex and striatum displayed non-significant [^18^F]CPFPX PET signal changes at different days after MCAO (Figures 3D and F).

### Metabolite analyses of [^18^F]CPFPX after MCAO

**Figure 4:**
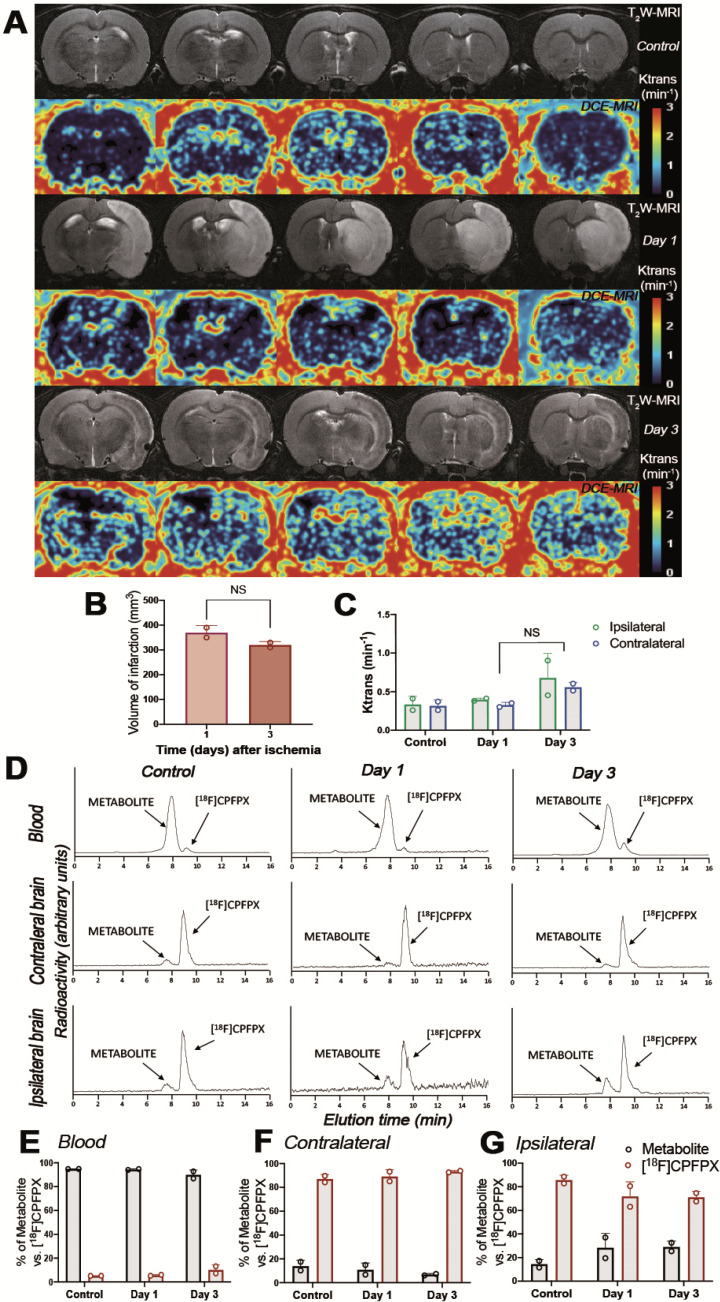
Magnetic resonance imaging (MRI) (T_2_-weighthing (T_2_W)), Dynamic Contrast Enhanced (DCE) images and Radio-HPLC before (day 0) and at days 1 and 3 after cerebral ischemia. MRI-T_2_W and DCE-MRI axial images show the ischemic lesion and blood brain barrier disruption (BBBd) evolution during first 3 days after stroke onset (A). Infarct volume with MRI (T_2_W) (B), and BBBd with DCE-MRI (C) were evaluated at control (n = 2) and at days 1 (n = 2) and 3 (n = 2) after MCAO in rats. Chromatograms show [^18^F]CPFPX and metabolite formation in blood, contralateral and ipsilateral brain hemispheres at 60 min after radiotracer injection (D). % of metabolite and parent compound was evaluated in same animals subjected to MRI in blood (E), contralateral (F) and ipsilateral (G) cerebral hemispheres. Infarct volume and BBBd values show non-significant changes at different days after MCAO. Values are presented as scatter dot blot (mean ± SD).

The evaluation of the parent compound ([^18^F]CPFPX) and its metabolites was carried out by radio-HPLC at 60 min after injection of [^18^F]CPFPX in both control and ischemic rats (Figure 4). Rats were subjected to T_2_W-MRI and DCE-MRI to evaluate the extension of the ischemic lesion and the BBBd at control and after ischemia (days 1 and 3), respectively, to evaluate the effect of BBB integrity on metabolite accumulation in ischemic brain (Figure 4A). These results showed a non-significant decrease of the volume of infarction at day 3 in relation to day 1 after MCAO (Figure 4B). Likewise, Ktrans values showed a non-significant increase of BBBd at day 3 in relation to control and day 1 after ischemia (Figure 4C). One major metabolite was detected as result of metabolism of [^18^F]CPFPX in both healthy and ischemic conditions with Radio-HPLC (Figure 4D). Both the metabolite and [^18^F]CPFPX were detected in blood and both brain hemispheres using the same rats subjected to MRI studies (Figure 4D). At 60 min after [^18^F]CPFPX administration, almost all the injected radiotracer was metabolized in blood at control (94.85% ± 0.07) and at days 1 (94.40% ± 0.56) and 3 (89.85% ± 4.17) after MCAO (Figure 4E). In contrary, this situation was reverted in the non-ischemic (contralateral) brain hemisphere showing a higher percentage of [^18^F]CPFPX in relation to metabolite at control (87% ± 3.81) and at days 1 (89.15% ± 5.73) and 3 (93.25% ± 0.92) after ischemia (Figure 4F). Finally, the ischemic hemisphere showed a slight decrease of [^18^F]CPFPX accumulation at day 1 (71.75% ± 12.37) and day 3 (71.05% ± 5.02) in relation to control rats (85.65% ± 4.03) (Figure 4G). Likewise, the metabolite content increased in the region of infarction at days 1 (28.25% ± 12.37) and 3 (28.95% ± 5.02) in relation to control (14.35% ± 4.03) values (Figure 4G). Overall, these results showed that the metabolite accumulation in the ischemic brain was influenced by the disruption of BBB after MCAO (Figure 4C and G).

### Expression of purinergic A_1_ARs in microglia and infiltrated leukocytes after MCAO

**Figure 5:**
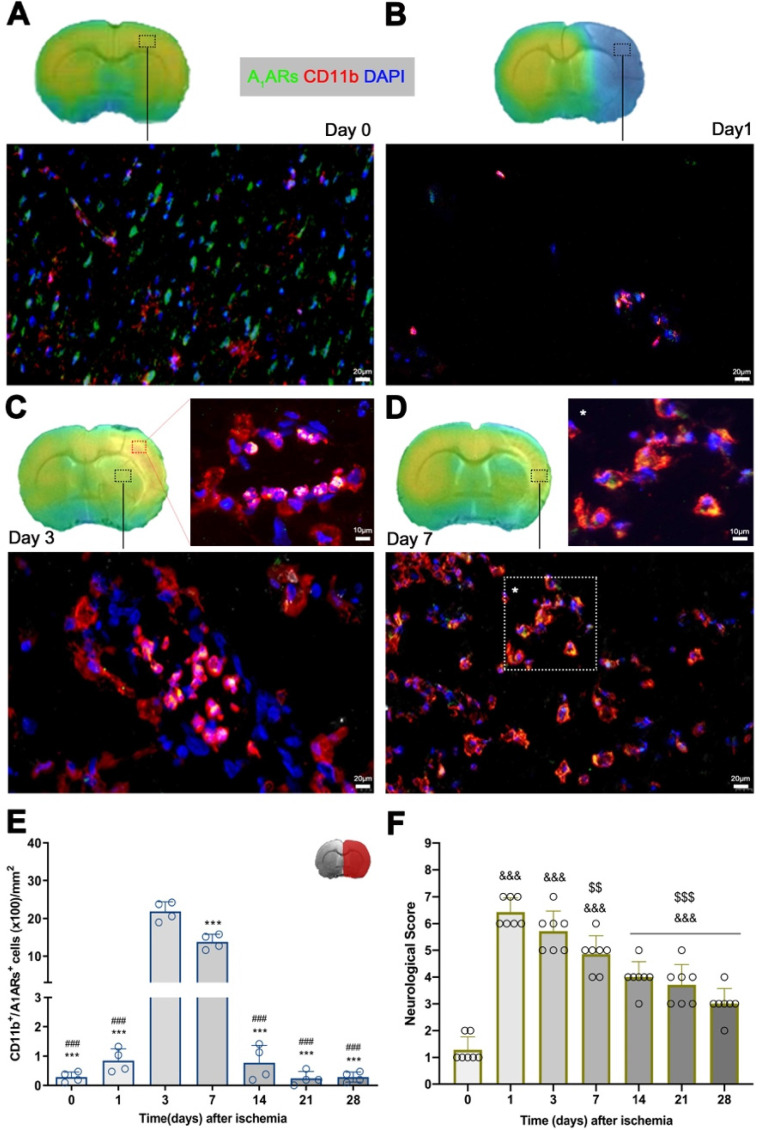
Immunofluorescent labeling of A_1_ARs (green), CD11b (red) and DAPI (blue) in the ischemic region, shown as merged channels and neurological outcome after ischemia. The data show the temporal evolution of A_1_ARs expression in microglia and infiltrated leukocytes (macrophages and neutrophils) expressing CD11b at day 0 (A), day 1 (B), day 3 (C) and day 7 (D) after ischemia. The number of CD11b-reactive microglia and infiltrated leukocytes (n = 4) were measured at different time points in the infarcted brain hemisphere (E). The upper right panel of this figure show the selected brain ROI for the quantification defined on an axial slice of a MRI (T_2_W) template. The neurological score show an improvement over time (F). ***p < 0.01 compared with day 3; ^###^*p* < 0.01 compared with day 7; ^&&&^*p* < 0.01 compared with day 0 and ^$$^*p* < 0.01 and ^$$$^*p* < 0.01 compared with day 1. Scale bars, 10 and 20 µm. Values are presented as scatter dot blot (mean ± SD).

Immunofluorescence staining showed A_1_ARs expression in a heterogeneous population of inflammatory cells such as microglia and infiltrated leukocytes (macrophages and neutrophils) after cerebral ischemia (Figure 5). At day 0, the expression of A_1_ARs was observed mainly in purinergic neurons distributed in different cerebral regions (Figure 5A), followed by a sharp decline at day 1 in the region of infarction (Figure 5B). At day 3, CD11b positive cells with round morphology and multilobulated nucleus (neutrophils) co-localized with the cellular expression of A_1_ARs (in yellow; Figure 5C). Finally, A_1_ARs expression in microglia/macrophage with ameboid shape expressing CD11b (in yellow; Figure 5D) was observed at day 7. Hence, the number A_1_ARs/CD11b cells displayed a sharp significant increase at days 3 to 7 in relation to days 0 and 1 followed by a decrease during the following weeks after reperfusion (*p* < 0.001, Figure 5E).

### Time course of neurologic score after experimental stroke

Neurofunctional impairment including sensory and motor deficits was evaluated with the 9-neuroscore test before (control) and at 1, 3, 7, 14, 21 and 28 days after MCAO (Figure 5F). Ischemic animals showed the major neurologic impairment at 1 day after MCAO in relation to day 0 (control animals) followed by a progressive functional recovery over time. The neurological impairment showed significant increase versus that in the controls at days 1, 3, 7, 14, 21, and 28 after ischemia (*p* < 0.001, Figure 5F). After day 3, rats showed a trend to a progressive functional recovery over time at days 7 (*p* < 0.01), 14, 21, and 28 (*p* < 0.001) in relation to day 1 after ischemia (Figure 5F).

### Effect of A_1_ARs modulation on stroke outcome and microglial/macrophage activation

**Figure 6:**
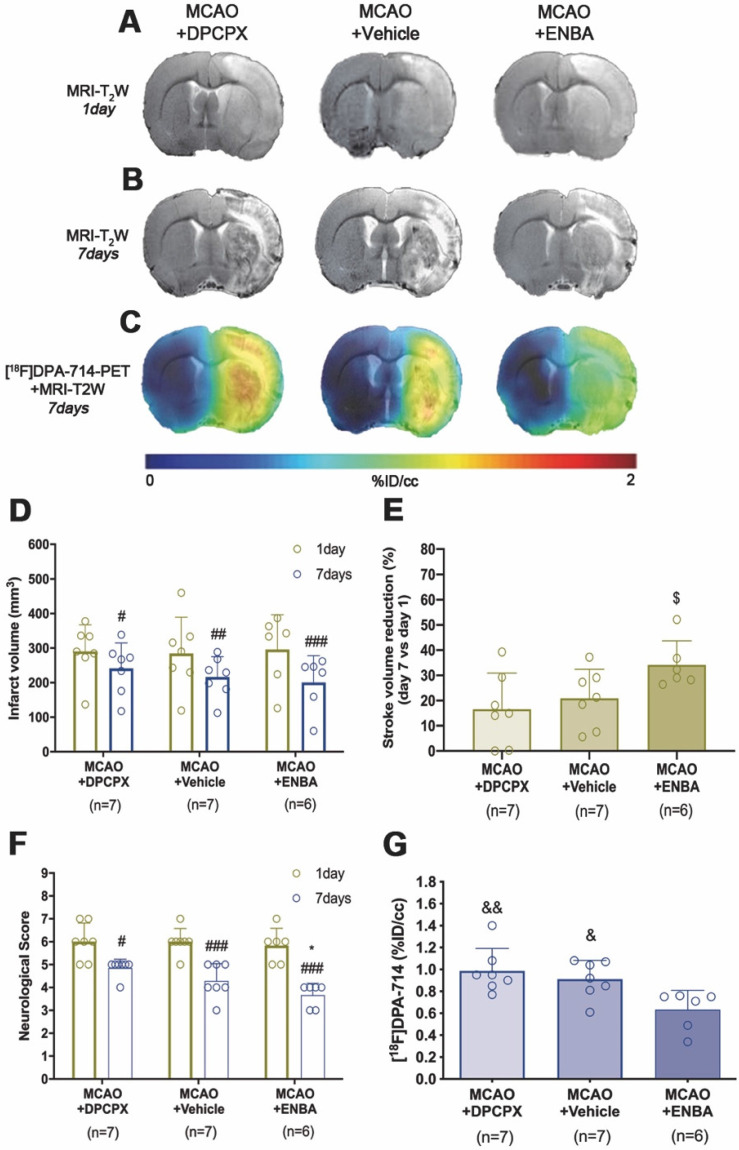
MRI-T_2_W and PET images of [^18^F]DPA-714 in DPCPX, vehicle and ENBA-treated ischemic rats. MRI (T_2_W) (A, B) and TSPO receptor PET signal (C) images of axial planes at the level of the ischemic lesion. Infarct volume with MRI (T_2_W) (D, E), neurological score (F) were evaluated at day 1 after ischemia (before the start of treatments) and at day 7 after MCAO in DPCPX (n = 7), vehicle (n = 7) and ENBA-treated (n = 6) rats. [^18^F]DPA-714 PET signal was quantified at day 7 after ischemia (G). *p < 0.05 compared with vehicle; ^#^*p* < 0.05, ^##^*p* < 0.01 and ^##^*p* < 0.001 compared with day 1; ^$^*p* < 0.05, compared with MCAO+DPCPX; ^&^*p* < 0.05 and ^&&^*p* < 0.01 compared with MCAO+ENBA. Values are presented as scatter dot blot (mean ± SD).

**Figure 7:**
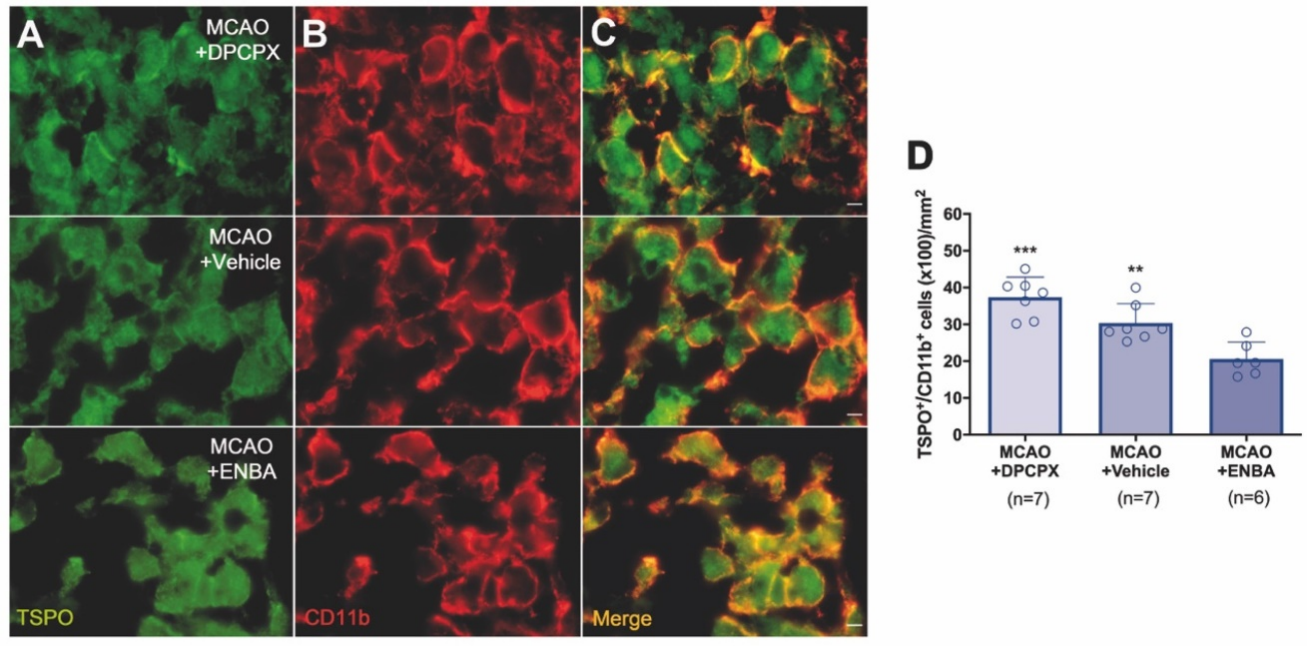
Immunofluorescent labeling of TSPO (green) and CD11b (red) in the ischemic area, shown as two channels. The data show TSPO expression in microglia/macrophages at day 7 after MCAO in DPCPX (upper row), vehicle (middle row), and ENBA-treated rats (lower row). TSPO expression (A) in CD11b-reactive microglia/macrophages (B) decreases after MCAO in ENBA-treated rats in merged images of two immunofluorescent antibodies (C). The number of CD11b-reactive microglia/macrophages expressing TSPO was evaluated at day 7 after daily treatment with DPCPX (n = 7), Vehicle (n = 7) and ENBA (n = 6) (D). **p < 0.01 and ***p < 0.01 compared with MCAO+ENBA. Scale bars, 5 µm. Values are presented as scatter dot blot (mean ± SD).

The role of A_1_ARs on stroke evolution and inflammatory reaction was explored using MRI, PET, behavioral evaluation and immunohistochemistry after daily treatment (from day 1 to day 7) after MCAO with the selective A_1_AR antagonist (DPCPX), the highly selective A_1_AR (ENBA) and vehicle after MCAO. Infarct volumes measured by T_2_W-MRI at day 1 after ischemia, before the starting of treatments, was not significantly different among different experimental groups (Figure 6D). Infarct volumes at day 7 showed a significant decrease in ischemic treated animals with DPCPX, Vehicle and ENBA (*p* < 0.05; *p* < 0.01; *p* < 0.001, Figure 6D) in comparison to day 1 (before the initiation of the treatments). Likewise, the activation of A_1_ARs with ENBA showed significant stroke infarction reduction at day 7 in relation to ischemic animals treated with DPCPX (*p* < 0.05, Figure 6E). Additionally, the different experimental groups of animals presented similar neurological impairment before the start of the treatments (day 1) (Figure 6F). One week later, the neurological outcome showed a significant improvement in relation to day 1 after different treatments (*p* < 0.05; *p* < 0.001, Figure 6F) in comparison to day 1 (before the initiation of the treatments). Besides, the pharmacological activation of A_1_ARs with ENBA after ischemia displayed a significant reduction of the neurological outcome relative to vehicle-treated ischemic rats at day 7 after MCAO (*p* < 0.05, Figure 6F). The effect of pharmacologically modulation of A_1_ARs on microglial/macrophage activation was explored with PET imaging of TSPO using the radiotracer [^18^F]DPA-714 at day 7 after MCAO. All images were quantified in standard units (%ID/cc). Axial images with normalized color scale showed [^18^F]DPA-714 PET signal differences in DPCPX (66.92 ± 13.79 MBq; 0.99 ± 0.59 nmol), Vehicle (72.10 ± 6.81 MBq; 0.84 ± 0.49 nmol) and ENBA (71.29 ± 9.04 MBq; 0.82 ± 0.52 nmol) treated ischemic rats (Figure 6C and Table S2). Treatment with ENBA rendered a significant decrease of PET signal in the ischemic cerebral hemisphere in comparison to DPCPX and Vehicle treated MCAO rats (*p* < 0.05; *p* < 0.01, Figure 6G). Immunofluorescence staining displayed TSPO over-expression (in green; Figure 7A) in CD11b^+^ cells after ischemia (in red; Figure 7B) in DPCPX, Vehicle and ENBA treated rats (Figure 7B). At day 7, TSPO over-expression co-localized with reactive microglia/macrophages showing intense CD11b immunoreactivity in the ischemic lesion (in green and red; Figure 7C). The number of TSPO^+^/CD11b^+^ cells showed a significant decrease in treated ischemic rats with ENBA at day 7 after ischemia in comparison with DPCPX and Vehicle treated rats (*p* < 0.01; *p* < 0.01, Figure 7D).

### Role of A_1_ARs modulation on microglial/macrophage proliferation

**Figure 8:**
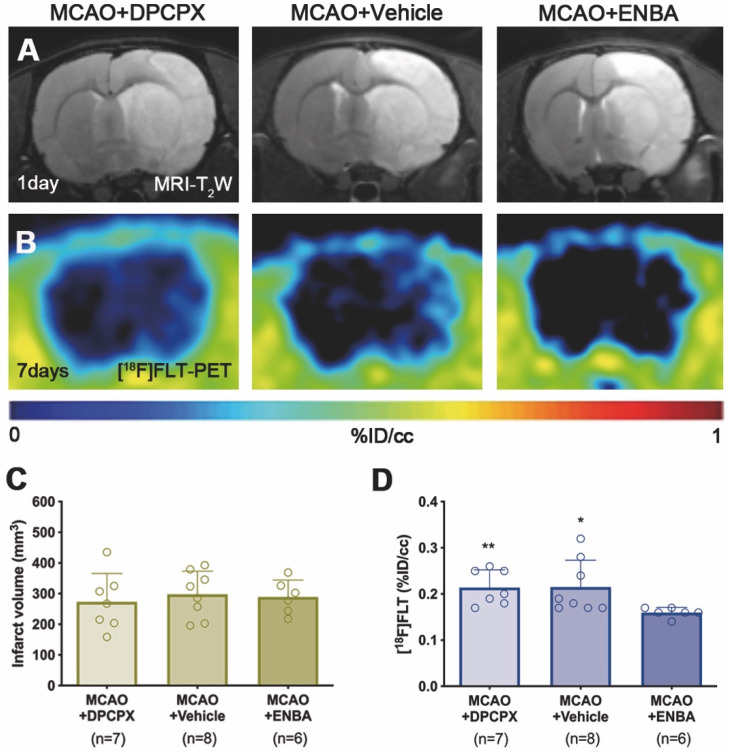
MRI-T_2_W and PET images of [^18^F]FLT in DPCPX, vehicle and ENBA-treated ischemic rats. MRI (T_2_W) (A) and glial proliferation PET signal (B) images of axial planes at the level of the ischemic lesion. Infarct volume with MRI (T_2_W) (C) at day 1 and [^18^F]FLT PET uptake (D) at day 7 after MCAO in DPCPX (n = 7), vehicle (n = 8) and ENBA-treated (n = 6) rats. ^*^p < 0.05, ^**^p < 0.01 compared with MCAO+ENBA. Values are presented as scatter dot blot (mean ± SD).

**Figure 9:**
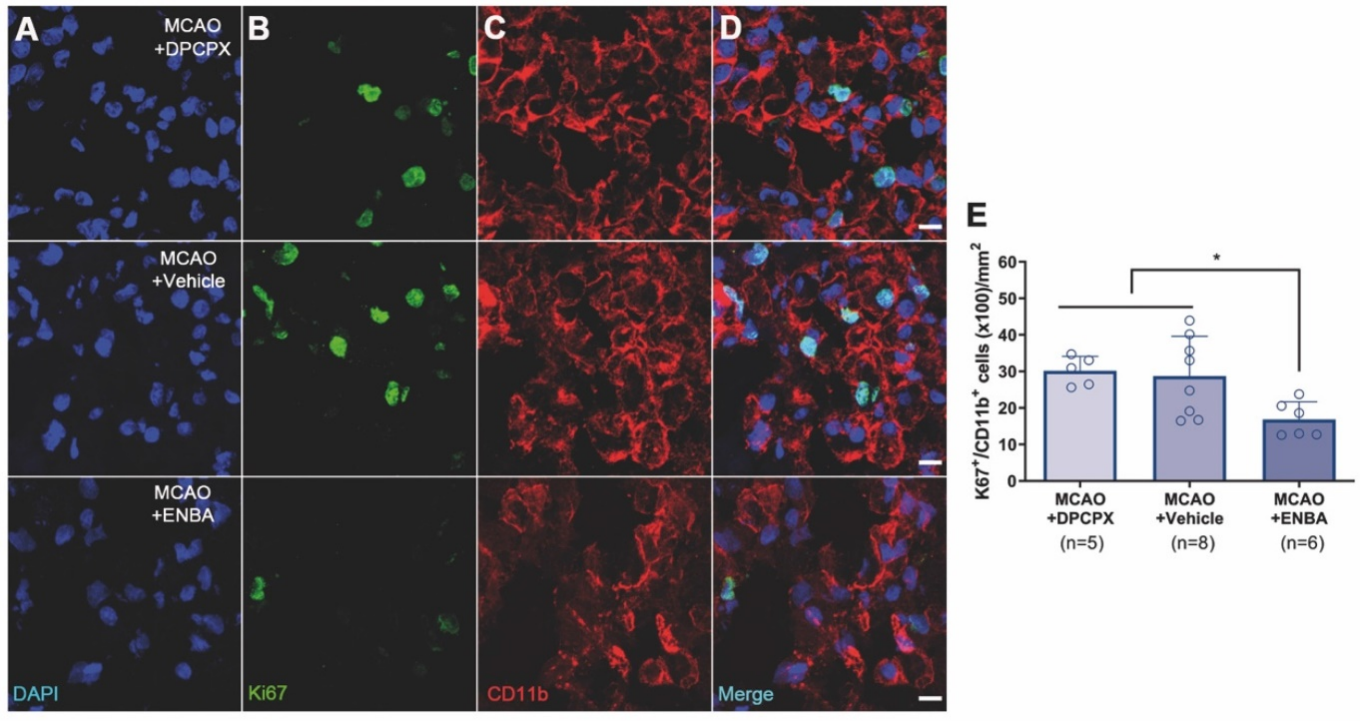
Immunofluorescent labeling of DAPI (blue), Ki67 (green) and CD11b (red) in the ischemic region, shown as three channels. The data show proliferative (Ki67^+^), microglia/macrophages (CD11b^+^) at day 7 after MCAO in DPCPX (upper row), vehicle (middle row), and ENBA-treated rats (lower row). Proliferative CD11b-reactive microglia/macrophages decrease after MCAO in ENBA-treated rats (see merged images) (D). The number of CD11b-reactive microglia/macrophages showing positive Ki67 was evaluated at day 7 after daily treatment with DPCPX (n = 5), Vehicle (n = 8) and ENBA (n = 6) (E). *p < 0.05 compared with MCAO+ENBA. Scale bars, 10 µm. Values are presented as scatter dot blot (mean ± SD).

The effect of A_1_ARs modulation on the proliferation of microglia and infiltrated macrophages was evaluated using PET and immunohistochemical studies after treatments with DPCPX, Vehicle and ENBA during the first week after MCAO. T_2_W-MRI evaluation showed similar infarct volumes at day 1 after ischemia (before the start of treatments) among different experimental groups to avoid bias (Figure 8A and C). The effects of the pharmacological modulation of A_1_ARs on the proliferation of microglial and infiltrated macrophages was evaluated using PET with the radiotracer [^18^F]FLT at day 7 after MCAO. Normalized PET images showed [^18^F]FLT PET signal in the region affected by the cerebral infraction in ischemic rats treated with DPCPX (52.84 ± 4.35 MBq) and Vehicle (53.66 ± 2.70 MBq) that was significantly decreased in ENBA (57.55 ± 2.19 MBq) treated rats at day 7 after MCAO (p < 0.05; p < 0.01, Figures 8B, 8D and Table S3). Immunohistochemical studies showed proliferative CD11b^+^ cells (in green-Ki67 and red-CD11b; Figures 9B and C) in DPCPX, Vehicle and ENBA ischemic treated rats. At day 7, ENBA treated animals displayed a significant reduction in the number of proliferating (Ki67^+^) microglia/macrophages in comparison with DPCPX and Vehicle treated rats (*p* < 0.05, Figure 9D and E).

## Discussion

The present study showed the overexpression of A_1_ARs during neuroinflammation underlying experimental stroke using PET imaging with [^18^F]CPFPX and immunohistochemical studies. Moreover, the therapeutic modulation of A_1_ARs improved stroke outcome and declined both the activation and proliferation of microglia and macrophages with [^18^F]DPA-714 and [^18^F]FLT radioligands, respectively.

### *In vivo* and *ex vivo* overexpression of A_1_ARs after experimental stroke

The *in vivo* distribution of [^18^F]CPFPX binding in the healthy human and rats brains were characterized by the distribution pattern of A_1_ARs in grey matter regions such as cortex and striatum, among others 11, 12. Hence, previously reported findings are consistent with the [^18^F]CPFPX PET uptake distribution in the healthy rat brain (before the induction of cerebral ischemia) observed in our work (day 0; Figure 2). In addition, metabolite analysis showed circa 90% of parent compound ([^18^F]CPFPX) in the healthy brain at 60 min after radioligand injection evidencing the stability and usefulness of [^18^F]CPFPX for *in vivo* PET imaging studies (Figure 4).

During the first stages after ischemia, [^18^F]CPFPX PET signal displayed a significant decrease as a direct consequence of the massive neuronal death process during stroke (day 1; Figure 2). In fact, these results support those described by Nariai and colleagues who observed that the degree of decreased PET binging to A_1_ARs was a sensitive predictor of severe ischemic insult 32. As a response to cellular stress during hypoxia and inflammation, adenosine controls pro-inflammatory and anti-inflammatory responses through A_1_ARs expressed in microglia and infiltrated macrophages in the central nervous system 33. In addition, [^18^F]CPFPX PET imaging detected peritumoral changes in A_1_ARs density concomitantly with the high activation of glial cells in the vicinity of the glioma 34. In the present study, A_1_AR expression with [^18^F]CPFPX was evaluated during the following month after MCAO in rats, to examine its relation with the neuroinflammatory reaction after cerebral ischemia. In the ischemic territory, [^18^F]CPFPX uptake showed up a sharp significant increase at days 3 and 7 in relation to day 1 as a response to neuroinflammation followed by a progressive reduction of A_1_ARs expression from days 14 to 28 after ischemia due to cell death and resolution of the inflammatory response (Figures 2 and 3). Additionally, metabolite studies in ischemic rats at days 1 and 3 after MCAO showed similar cerebral accumulation of both [^18^F]CPFPX and metabolite despite PET imaging studies showed opposite uptake values at day 1 in relation to day 3 after ischemia (Figures 2 to 4). Hence, A_1_ARs expression changes observed with [^18^F]CPFPX PET cannot be attributed to radiotracer metabolization following cerebral ischemia.

Altogether, these findings are in agreement with previous PET studies of cholinergic (α7 and α4β2 receptors) and glutamatergic (system xc^-^) signaling which showed overexpression of these receptors on glial cells during the first week after ischemia 27. Therefore, altogether might suggest that [^18^F]CPFPX PET signal increase is linked to the neuroinflammatory reaction underlying stroke. To verify this hypothesis, the immunohistochemical characterization of A_1_ARs on glial cells was carried out during the following month after MCAO (Figure 5). These results confirmed the overexpression of A_1_ARs in microglia and infiltrated macrophages (macrophages and neutrophils) at days 3 to 7 after MCAO. Therefore, these findings supported the evidence that the [^18^F]CPFPX signal increase after cerebral ischemia was mainly due to the A_1_ARs over-expression in CD11b positive cells (microglia and infiltrated macrophages). Likewise, the peak of A_1_ARs expression observed by PET imaging from day 3 to 7 was consistent with the functional recovery observed in the ischemic rats (Figure 5F). Additionally, the profile of A_1_AR expression was in agreement with the temporal expression of some anti-inflammatory markers and cytokines such as CD206, YM1/2 and Arg1 after cerebral ischemia, as previously described by Hu and colleagues 35.

### A_1_ARs activation improves stroke outcome and attenuates inflammatory activation

Previously, the pharmacological activation of A_1_ARs with selective agonists has shown therapeutic potential attenuating ischemic brain damage after animal models of stroke 36, 37. In addition, the activation of these receptors increased the IL10 expression whereas KO mice lacking A_1_ARs have a reduced interleukin expression by immune cells after neonatal hypoxic ischemic brain injury 7. In our study, we evaluated the modulatory effect of the antagonist DPCPX and the agonist ENBA on stroke outcome with MRI-T_2_W and neuroinflammatory reaction with [^18^F]DPA-714 PET following cerebral ischemia (Figure 6A to C). We showed a beneficial effect of ENBA on brain edema formation and neurofunctional evolution in relation to vehicle and DPCPX treated ischemic rats (Figure 6D to F). The improvement of stroke outcome at day 7 after daily treatment with ENBA was concomitant with the significant decrease of TSPO expression with [^18^F]DPA-714, supporting the role played by A_1_ARs on neuroinflammation after cerebral ischemia (Figure 6G). Besides, ischemic rats treated with ENBA showed a significant decrease of CD11b positive cells expressing TSPO (Figure 7), confirming the results obtained with PET imaging using [^18^F]DPA-714. In fact, this is the first study using PET imaging to evaluate the modulatory effect of A_1_ARs on neuroinflammation after ischemia using TSPO as biomarker of reactive gliosis. Therefore, our results were supported by those studies showing the role of A_1_ARs in the morphological activation of microglia playing a tight control in microglia physiology 38, 39. In this sense, the chronic ingestion of caffeine, a well-known antagonist for A_1_ARs, displayed a systemic impact on the activation of microglia but not on their proliferation in the healthy brain 39. In contrast, other studies confirmed the increase of microglial proliferation after simultaneous stimulation of both A_1_ and A_2A_ receptors 40. Thus, we evaluated the effect of A_1_AR modulation on glial proliferation with PET imaging after stroke to better answer this question.

### Effect of A_1_ARs modulation on microglia/macrophage proliferation after ischemia

Recently, we described a novel imaging method to quantify *in vivo* glial proliferation using PET with [^18^F]FLT 21. This radiotracer is an analog of thymidine which is phosphorylated by thymidine kinase-1 (TK-1), an enzyme expressed during the DNA synthesis and up-regulated during the S phase of the cell cycle 41. This study showed [^18^F]FLT signal increase in the ischemic lesion during the first week after MCAO that was in accordance with the increase of proliferative microglia/infiltrated macrophages 21. In the current study, we have assessed the effect of A_1_ARs modulation with DPCPX and ENBA on microglia/macrophage proliferation using [^18^F]FLT PET after stroke (Figure 8). The activation of A_1_ARs with ENBA showed a significant decrease of cellular proliferation with [^18^F]FLT PET at day 7 after MCAO (Figure 8D). In addition, ischemic rats treated with ENBA showed a significant decrease of proliferative microglia/infiltrated macrophages (CD11b positive cells) expressing Ki67, a well-known marker for the evaluation of proliferation 42 (Figure 9), confirming the results obtained with PET imaging using [^18^F]FLT.

## Summary and Conclusions

In summary, multimodal imaging studies were carried out to decipher the role of A_1_ARs on neuroinflammatory reaction after cerebral ischemia in rats. The present findings showed that [^18^F]CPFPX PET signal increases in the ischemic hemisphere at day 3 after ischemia followed by a progressive decline afterwards. In addition, A_1_AR overexpression was identified in microglia and infiltrated leukocytes using immunohistochemistry. Finally, the activation of A_1_AR was able to induce ischemic damage protection and reduction of both reactive and proliferative microglia/macrophages after experimental stroke in rats. Altogether, our results provide novel knowledge regarding the control of A_1_AR on ischemic damage and inflammation that might contribute to the development of novel therapies for stroke.

## Supplementary Material

Supplementary tables.
