# Supplementary material for: In vivo multimodal imaging of adenosine A1 receptors in neuroinflammation after experimental stroke
Source: Theranostics. 2021 Jan 1;11(1):410–25. doi: 10.7150/thno.51046 (PMC7681082; doi:10.7150/thno.51046)
Supplement: Supplementary file 1 — Supplementary tables. [file thnov11p0410s1.pdf]

Supplementary material

[18F]CPFPX IA/IM	#1	#2	#3	#4	#5	#6	#7	AVG
**D0**	53.62/ 0.11	73.26/ 0.15	76.30/ 0.16	74.41/ 0.25	63.20/ 0.33	64.01/ 0.54	68.82/ 1.08	67.80±8.14/ 0.37±0.34
**D1**	76.22/ 0.17	39.90/ 0.19	72.52/ 0.26	72.89/ 0.46	63.64/ 0.64	72.15/ 0.79	54.76/ 0.88	65.25±13.11/ 0.47±0.29
**D3**	81.31/ 0.15	77.33/ 0.16	67.71/ 0.23	68.45/ 0.37	61.42/ 0.53	74.37/ 1.03	62.16/ 1.39	64.48±/7.55 0.56±0.48
**D7**	55.13/ 0.11	68.08/ 0.24	76.59/ 0.32	68.45/ 0.40	72.15/ 0.74	76.96/ 1.26	64.01/ 1.68	68.77±/7.62 0.68±0.58
**D14**	80.17/ 0.28	61.79/ 0.13	63.64/ 0.22	61.79/ 0.35	60.68/ 0.55	69.93/ 1.08	66.60/ 1.70	66.37±/6.89 0.61±0.57
**D21**	70.67/ 0.16	68.08/ 0.17	66.60/ 0.27	35.52/ 0.32	67.71/ 0.40	66.97/ 1.11	55.50/ 1.48	61.58±/12.46 0.56±0.52
**D28**	69.19/ 0.15	62.90/ 0.21	72.52/ 0.26	70.67/ 0.36	66.60/ 0.38	63.64/ 0.59	68.82/ 1.76	67.76±/3.56 0.53±0.56

Table S1. Injected activity (IA, MBq) and injected mass (IM, nmol) doses for PET studies of [18F]CPFPX before and different days after cerebral ischemia in rats. Average data are presented as mean±SD.

[18F]DPA-714 IA/IM	#1	#2	#3	#4	#5	#6	#7	AVG
**DPCPX**	81.03/ 0.26	74.37/ 0.37	79.55/ 0.69	67.71/ 0.88	62.16/ 1.40	62.90/ 1.52	40.70/ 1.80	66.92±/13.79 0.99±0.59
**Vehicle**	69.19/ 0.21	79.55/ 0.48	75.85/ 0.61	62.90/ 0.88	71.78/ 0.90	80.29/ 1.03	65.12/ 1.77	72.10±/6.81 0.84±0.49
**ENBA**	87.69/ 0.42	66.23/ 0.43	75.85/ 0.50	68.45/ 0.57	64.75/ 1.43	64.75/ 1.54	N/A	71.29±/9.04 0.82±0.52

Table S2. Injected activities (IA, MBq) and injected mass (IM, nmol) doses for PET studies of [18F]DPA-714 in DPCPX, vehicle and ENBA-treated ischemic rats. Average data are presented as mean±SD.

[18F]FLT IA	#1	#2	#3	#4	#5	#6	#7	#8	AVG
**DPCPX**	54.60	57.22	59.57	49.38	51.37	49.41	48.33	N/A	52.84±4.35
**Vehicle**	54.22	49.82	50.84	51.45	56.11	57.50	55.23	54.10	53.66±2.70
**ENBA**	60.81	55.22	58.1	54.92	57.91	58.36	N/A	N/A	57.55±2.19

Table S3. Injected activity (IA, MBq) doses for PET studies of [18F]FLT in DPCPX, vehicle and ENBA-treated ischemic rats. Average data are presented as mean±SD.
